# Hat die SARS-CoV-2-Pandemie die Lehre verbessert? – Virtueller Unterricht im Fach HNO-Heilkunde aus Sicht der Studierenden

**DOI:** 10.1007/s00106-022-01192-8

**Published:** 2022-06-30

**Authors:** Alexa Krambeck, Andreas G. Loth, Martin Leinung, Anwar Syed-Ali, Natalie Filmann, Sabine Kramer, Uwe Baumann, Timo Stöver, Marc Diensthuber

**Affiliations:** 1 Klinik für Hals-Nasen-Ohrenheilkunde, Universitätsklinikum Frankfurt, Goethe-Universität, Theodor-Stern-Kai 7, 60590 Frankfurt am Main, Deutschland; 2 Dekanat des Fachbereichs Medizin, Universitätsklinikum Frankfurt, Goethe-Universität, Theodor-Stern-Kai 7, 60590 Frankfurt am Main, Deutschland; 3 Institut für Biostatistik und Mathematische Modellierung, Universitätsklinikum Frankfurt, Goethe-Universität, Theodor-Stern-Kai 7, 60590 Frankfurt am Main, Deutschland; 4 Schwerpunkt Phoniatrie und Pädaudiologie, Klinik für Hals-Nasen-Ohrenheilkunde, Universitätsklinikum Frankfurt, Goethe-Universität, Theodor-Stern-Kai 7, 60590 Frankfurt am Main, Deutschland; 5 Schwerpunkt Audiologische Akustik, Klinik für Hals-Nasen-Ohrenheilkunde, Universitätsklinikum Frankfurt, Goethe-Universität, Theodor-Stern-Kai 7, 60590 Frankfurt am Main, Deutschland

**Keywords:** Medizinstudium, Digitalisierung, Distanzunterricht, COVID-19, Lehrveranstaltungsevaluation, Medical education, Digitization, Distance learning, COVID-19, Course evaluation

## Abstract

**Hintergrund und Fragestellung:**

Die Severe acute respiratory syndrome coronavirus type 2(SARS-CoV-2)-Pandemie hat die Ausbildung von Medizinstudierenden grundlegend verändert. Die Notwendigkeit von Kontaktbeschränkungen und die damit einhergehende Forderung nach Distanzunterricht hat dazu geführt, dass innerhalb kurzer Zeit digitale Lehrformate umgesetzt werden mussten. Ziel dieser Arbeit war die Auswertung der studentischen Evaluationsergebnisse für virtuellen Unterricht im Fach Hals-Nasen-Ohren-Heilkunde während der SARS-CoV-2-Pandemie und ein Vergleich mit den zuvor erhobenen Evaluationsergebnissen unter Präsenzbedingungen.

**Material und Methoden:**

Untersucht wurden die Evaluationsergebnisse für die Blockpraktika im Wintersemester 2020/21 und im Sommersemester 2021, die in einem virtuellen Format mit kurzer Präsenzphase durchgeführt wurden, sowie die der komplett im konventionellen Präsenzformat durchgeführten Praktika von Sommersemester 2018 bis Wintersemester 2019/20. Die anonyme Befragung der Studierenden bezog sich auf verschiedene Aspekte der Lehrveranstaltung, wie z. B. Organisation, Didaktik und Lernatmosphäre.

**Ergebnisse:**

Von  16 abgefragten Kategorien zeigten 14 (87,5%) signifikant bessere Evaluationsergebnisse für die virtuellen Praktika verglichen mit den zuvor im Präsenzformat durchgeführten Praktika. Diese sehr positive Bewertung des digitalen Lehrangebots zeigte im Pandemieverlauf über die Dauer von zwei Semestern keine signifikante Änderung.

**Schlussfolgerung:**

Die vorliegenden Daten belegen die hohe Akzeptanz eines digitalen Lehrangebots im Fach HNO-Heilkunde für Studierende. Auch wenn unerlässliche Bestandteile der ärztlichen Ausbildung, wie der Unterricht am Patienten und das Erlernen klinisch-praktischer Fertigkeiten, weiterhin nur im Präsenzformat realisiert werden können, legen die Ergebnisse nahe, dass digitale Elemente auch nach der SARS-CoV-2-Pandemie eine Rolle im Medizinstudium spielen könnten.

## Abstract (en)

**Background:**

The Severe acute respiratory syndrome coronavirus type 2 (SARS-CoV‑2) pandemic has significantly changed the education of medical students. Due to the contact restrictions and the associated requirement for distance learning, digital teaching formats had to be implemented within a short period of time. The aim of our work was to analyze student evaluation data for virtual teaching in otorhinolaryngology (ORL) during the SARS-CoV‑2 pandemic and to compare the data with previously obtained evaluation data under face-to-face conditions.

**Materials and methods:**

Evaluation data for the block practical courses in winter semester 2020/21 and summer semester 2021, which were carried out in a virtual format with a short face-to-face phase as well as those for the block practical courses from summer semester 2018 to winter semester 2019/20, which had been performed completely in a conventional face-to-face format, were analyzed. The anonymous survey of the students focused on various aspects of the courses such as organization, didactics and learning atmosphere.

**Results:**

Of 16 surveyed categories, 14 (87.5%) showed significantly better evaluation results for the virtual courses compared to the courses carried out previously under face-to-face conditions. This very positive assessment of the digital teaching offer showed no significant change during the course of the pandemic over the period of two semesters.

**Conclusions:**

Our data show a high acceptance of digital teaching in ORL for students. Even though essential components of the medical education such as teaching on the patient and clinical-practical skills can still only be realized in a face-to-face format, our data suggest that digital elements could also play a role in medical education after the SARS-CoV‑2 pandemic.

## Hintergrund und Fragestellung

Die Severe acute respiratory syndrome coronavirus type 2(SARS-CoV-2)-Pandemie stellt eine globale Gesundheitskrise enormen Ausmaßes dar und hat bisher rund 5,9 Mio. Todesopfer gefordert [9]. Ein Blick zurück in die Vergangenheit zeigt jedoch, dass Pandemien oft auch Motor plötzlich notwendiger Veränderungen waren. So wie bereits in den „Goldenen Zwanziger Jahren“ („roaring twenties“) nach der Spanischen Grippe (1918–1920) eine Ära des Aufbruchs mit boomender Wirtschaft, aber vor allem auch bedeutenden technologischen Fortschritten folgte, könnte möglicherweise auch infolge der SARS-CoV-2-Pandemie ein ähnlicher Innovationsschub resultieren [2].

Die akademische Lehre hat sich bereits während der SARS-CoV-2-Pandemie grundlegend gewandelt und technologisch weiterentwickelt. Während E‑Learning in der Hochschullandschaft zumindest im Fach Hals-Nasen-Ohren-Heilkunde noch vor wenigen Jahren nur sehr begrenzt zum Einsatz kam [4, 7], gaben nahezu alle deutschen HNO-Universitätskliniken in einer strukturierten online-Befragung an, auf die Einschränkung der Präsenzlehre zu Beginn der SARS-CoV-2-Pandemie mit der Implementierung neuer Lehrformate reagiert zu haben [15]. Dies stellte zunächst eine erhebliche Herausforderung dar, denn – wie eine weitere Umfrage ergab – waren zu Beginn des Sommersemesters 2020 sowohl Universitätskliniken als auch akademische Lehrkrankenhäuser nur bedingt auf die Digitalisierung der Lehre vorbereitet [11].

Der Fachbereich Medizin des Universitätsklinikums Frankfurt startete zu dieser Zeit eine Initiative zur Förderung von Projekten zur Realisierung der Lehre nach den Richtlinien des Robert Koch-Instituts (RKI). In diesem Rahmen wurde auch die Umsetzung des Konzepts eines „interaktiv-integrativ-digitalen HNO-Praktikums“ unterstützt. Im Fokus dieses Lehrprojekts stand die Etablierung eines online-synchronen Distanzunterrichtformats, das durch den Einsatz von Lehrvideos, computerbasierten Lernprogrammen und Online-Seminaren das HNO-Praktikum für Medizinstudierende in Präsenz während der pandemiebedingten Kontaktbeschränkungen ersetzen sollte. Die neue Unterrichtsform kam während der Pandemie in zwei Semestern (Wintersemester 2020/21 und Sommersemester 2021) zum Einsatz.

Dieser Untersuchung lagen die Hypothesen zugrunde, dass ein Praktikum im Fach HNO-Heilkunde in einem virtuellen Distanzunterrichtsformat von Studierenden als „Notprogramm“ mit deutlich eingeschränkter Lehrqualität empfunden wird und dass die Akzeptanz der Studierenden für Distanzunterricht mit zunehmender Dauer der Pandemie abnimmt. Das Ziel der hier präsentierten Arbeit war daher die Beantwortung der Frage, wie Studierende dieses neue Unterrichtsformat im Pandemieverlauf bzw. im Vergleich zu konventionellem Präsenzunterricht in der präpandemischen Zeit bewerten.

## Material und Methoden

### Untersuchungszeitraum

Die Untersuchung umfasste die Blockpraktika im Fach HNO-Heilkunde in der Zeit vom Sommersemester 2018 bis zum Sommersemester 2021 (*n* = 6 Praktika). Das einwöchige Blockpraktikum (Mo–Fr) wurde in jedem Semester über einen Zeitraum von rund drei Monaten angeboten.

Bis zum Wintersemester 2019/20 wurde das Blockpraktikum in Form einer Präsenzveranstaltung durchgeführt. Während der SARS-CoV-2-Pandemie wurde erstmals ein virtuelles Praktikum angeboten. Für diese Untersuchung wurden die Evaluationsergebnisse von vier Semestern mit Präsenzunterricht (Sommersemester 2018 bis Wintersemester 2019/20; *n* = 4 Praktika) ausgewertet und mit den Evaluationsergebnissen der Praktika im virtuellen Format (Wintersemester 2020/21 und Sommersemester 2021; *n* = 2 Praktika) verglichen. Im Sommersemester 2020 konnte das Blockpraktikum aufgrund der pandemischen Lage (erste Welle) nicht durchgeführt werden, weshalb für dieses Semester keine Evaluationsdaten vorlagen.

### Konzept des konventionellen HNO-Praktikums im Präsenzformat vor Beginn der SARS-CoV-2-Pandemie

Das bis zum Wintersemester 2019/20 durchgeführte, einwöchige HNO-Blockpraktikum umfasste einen HNO-Spiegelkurs, einen Kopf-Hals-Sonographie-Kurs, einen Audiologie‑/Vestibulariskurs (mit computerbasierter Simulation einer tonaudiometrischen Untersuchung, Simulation eines Ausfalls des Gleichgewichtsorgans), eine Hospitation mit Unterricht am Patienten in der Hochschulambulanz, eine Hospitation in den Operationssälen der Klinik, eine Lehrvisite auf den Stationen, einen Untersuchungskurs mit Patientenvorstellung, einen Phoniatrie- und Pädaudiologiekurs sowie verschiedene Seminare, die der praxisorientierten Wissensvermittlung dienten (z. B. Management von Notfällen in der HNO-Heilkunde). Alle praktischen Unterrichtseinheiten wurden in Kleingruppen à 3–4 Studierende durchführt.

### Konzept des interaktiv-integrativ-digitalen HNO-Praktikums während der SARS-CoV-2-Pandemie

Um das Praktikum im Fach HNO-Heilkunde unter Einhaltung der RKI-Richtlinien zu realisieren, wurde im Juni 2020 das Konzept eines „interaktiv-integrativ-digitalen HNO-Praktikums“ entwickelt. Das Ziel des Lehrkonzepts war es, *digitale* Lehrinhalte zu erstellen, in die inhaltliche Struktur des bestehenden Praktikums zu *integrieren* und mit dem online-synchron durchgeführten Unterricht den *interaktiven* Aspekt (Dozent/Studierende) der Lehrveranstaltung zu gewährleisten. Hierfür wurden Drehbücher für Lehrvideos (z. B. Spiegelkurs, audiometrische/neurootologische Verfahren, HNO-ärztliche Operationen) erarbeitet, die dann mit Unterstützung der Abteilung für Medienproduktion der Zentralen eLearning-Einrichtung „studiumdigitale“ der Goethe-Universität in der HNO-Klinik angefertigt wurden. Diese Videos wurden schließlich unter Verwendung des Videokonferenzsystems Zoom (Zoom Video Communications Inc., San Jose, USA) im Rahmen von online-synchron durchgeführten Seminaren gestreamt und von einem Dozenten/Moderator kommentiert und diskutiert.

Für das praktische Erlernen audiometrischer Testverfahren wurde im zweiten virtuellen Semester (Sommersemester 2021) außerdem eine Remote-Nutzung des computerbasierten Audiometrie-Trainingsprogramms „Otis“ (Innoforce Est., Ruggell, Liechtenstein) über die Fernsteuerungsfunktion von Zoom vor Beginn der Kurseinheiten eingerichtet. Hierzu wurden die Studierenden zunächst mit den Grundlagen der Tonaudiometrie vertraut gemacht und nach einer kurzen Einführung in die Bedienung des virtuellen Audiometers in Kleingruppen aus 2–3 Personen in bis zu 6 „Break-out“-Räume verteilt. Die Aufgabe der Kleingruppe bestand darin, nach Durchführung einer virtuellen Ohrinspektion und des Weber‑/Rinne-Tests verschiedene Hörstörungen (beidseitige kombinierte Schwerhörigkeit, asymmetrischer Innenohrhörverlust, akuter Hörsturz) in Luft- und Knochenleitung zu audiometrieren, die jeweiligen Schwellen korrekt zu erfassen und eine Diagnose der vorliegenden Hörstörung zu stellen. Zur Unterstützung der parallel arbeitenden Gruppen konnte sich bei auftretenden Fragen oder Problemen ein Tutor in die jeweilige Gruppe hinzuschalten, um entsprechende Hilfen zu geben.

Der online-synchrone Modus der virtuellen Lehrveranstaltung ermöglichte über die gesamte Dauer des Praktikums die Interaktion zwischen Dozent und Studierenden, die von zuhause aus mit einem digitalen Endgerät (Computer, Tablet oder Smartphone) an der Lehrveranstaltung teilnahmen. Ergänzt wurde das virtuelle Praktikum durch eine vierstündige Präsenzphase, die aus einer Lehrvisite auf der Station und einer Hospitation in der Hochschulambulanz bestand und in Kleingruppen unter strikter Einhaltung der zum jeweiligen Zeitpunkt geltenden Hygienevorgaben erfolgte. Direkter Patientenkontakt fand angesichts des Infektionsrisikos für die Studierenden nur in reduziertem Umfang statt. Es handelte sich somit insgesamt um eine hybride Lehrveranstaltung. Da der zeitliche Umfang dieser Präsenzphase mit einer Dauer von 4 h jedoch nur einen sehr geringen Anteil an dem einwöchigen Praktikum hatte, wird in dieser Arbeit durchgängig die Bezeichnung „virtuell“ bzw. „digital“ für dieses Lehrformat verwendet.

### Lehrevaluation durch Medizinstudierende

Die studentische Lehrevaluation wurde durch das Studiendekanat des Fachbereichs Medizin unter Verwendung der Befragungssoftware evasys (evasys GmbH, Lüneburg, Deutschland) durchgeführt. Das Verfahren erfolgte anonym, und durch die Verwendung von TAN-Nummern konnte eine Mehrfachabstimmung ausgeschlossen werden.

**Figure 1:**
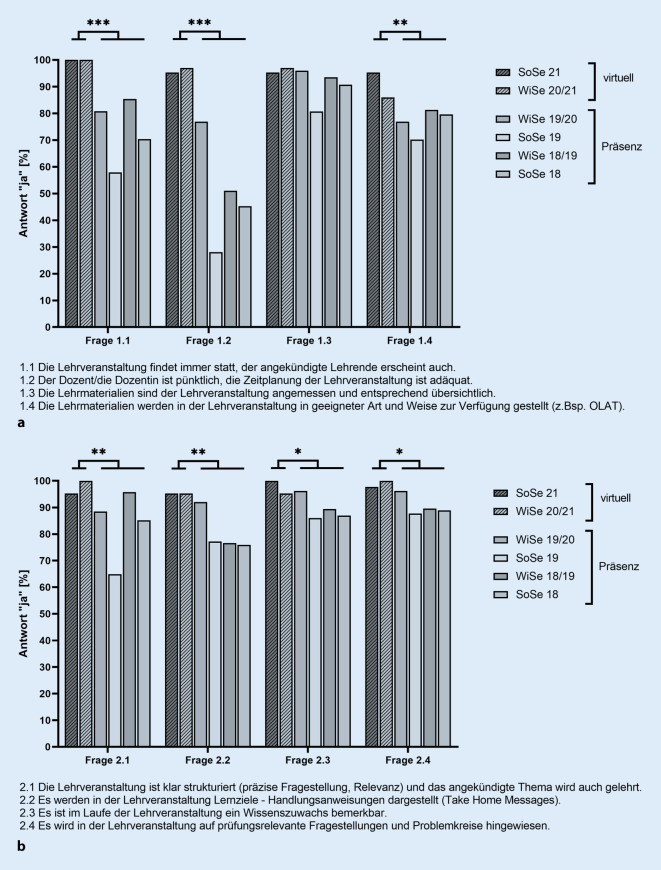

**Figure 2:**
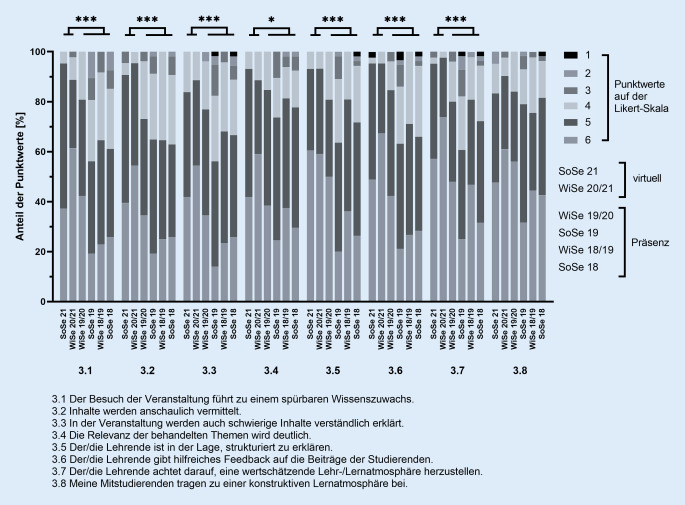

Die Studierenden wurden im Anschluss an das Blockpraktikum per E‑Mail zur freiwilligen Teilnahme an der Evaluation eingeladen. Der Fragebogen für die Evaluation umfasste insgesamt 16 geschlossene Fragen: 8 Fragen waren vom dichotomen Typ (Antwortmöglichkeiten: ja/nein) und 8 Fragen sollten anhand der Likert-Skala (Kategorien 1–6; 1: stimme überhaupt nicht zu, 6: stimme voll und ganz zu) beantwortet werden. Die Fragen waren thematisch den Bereichen „Organisation der Lehrveranstaltung“ (Fragen 1.1–1.4, Abb. 1a), „Didaktik der Lehrveranstaltung“ (Fragen 2.1–2.4, Abb. 1b) und weiteren „Angaben zur Lehrveranstaltung“ (Fragen 3.1–3.8, Abb. 2) zugeordnet.

**Figure 3:**
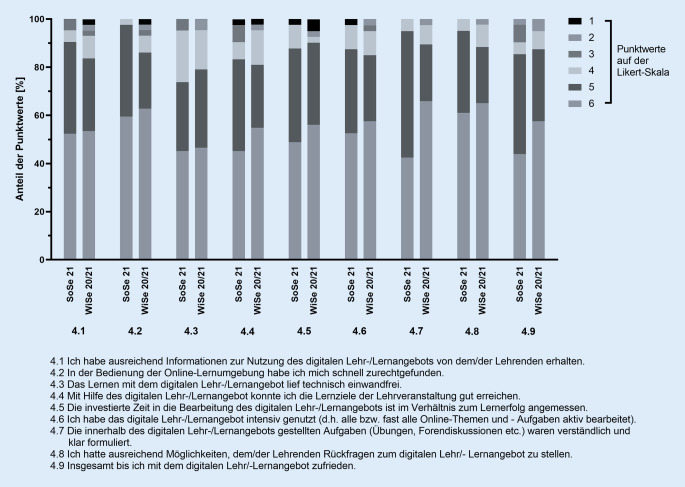

Der Fragebogen wurde mit Einführung des virtuellen Praktikums im Wintersemester 2020/21 um zusätzliche 9 Fragen erweitert, die ausschließlich auf die neuen, digitalen Lehrinhalte fokussierten (Abb. 3).

Im Rahmen der Evaluation gab es auch die Gelegenheit, offenes Feedback zur Lehrveranstaltung zu geben. Diese frei formulierten Rückmeldungen wurden in den virtuellen Semestern mit Fokus auf Aussagen mit dem Inhalt „Wunsch nach mehr Präsenzzeit/-unterricht“ quantitativ ausgewertet.

### Statistik

Die statistische Auswertung erfolgte mit R (R Core Team, 2020. R: A language and environment for statistical computing. R Foundation for Statistical Computing, Wien, Österreich. Verwendete Pakete: nlme, logistf). Zielgrößen waren die Antwortmöglichkeiten der Fragen des Fragebogens: ja/nein bei den Fragekomplexen F1, F2 (dichotome Fragen). Bei den Fragekomplexen F3, F4 (Likert-Skala) wurden die Antwortmöglichkeiten der ordinalen Skala für die Auswertung wie folgt dichotomisiert: ≤ 4 versus > 4. Einflussgröße für den Vergleich von virtuellen Praktika und Präsenzpraktika war die Unterrichtsform (d. h. virtuell/Präsenz). Dazu wurde eine logistische Regression mit zufälligen Effekten verwendet. Als zufälliger Effekt wurde hierbei das jeweilige Semester betrachtet, d. h. die Studierenden eines Semesters bildeten ein Cluster. Im Fall von Konvergenzproblemen wurde die logistische Regression ohne zufälligen Effekt verwendet und bei extremer Unbalanciertheit kam die Firth-Regression zur Anwendung. Zum Vergleich der beiden virtuellen Semester wurde eine logistische Regression verwendet. Alle *p*-Werte sind zweiseitig, und es wurde ein Signifikanzniveau von 5 % verwendet. Die grafische Darstellung der Evaluationsergebnisse erfolgte mit GraphPad Prism 9.3.1 (GraphPad Software, San Diego, CA, USA).

## Ergebnisse

### Rücklaufquote der Evaluation

Die Anzahl der im Untersuchungszeitraum an der Evaluation teilnehmenden Studierenden betrug 45,3 ± 11,0 und variierte zwischen *n* = 26 Teilnehmern im Wintersemester 2019/20 und *n* = 57 Teilnehmern im Sommersemester 2019. Die Rücklaufquote betrug 27,5 ± 6,0 %. Sie war am geringsten im Wintersemester 2019/20 (15,2 %) und am höchsten im Sommersemester 2019 (35,8 %).

### Evaluationsergebnisse: virtuelle Lehre vs. Präsenzlehre

#### Themenbereich „Organisation der Lehrveranstaltung“

Die Fragen zur Organisation bezogen sich auf die Regelmäßigkeit der Lehrveranstaltung (Frage 1.1), die Pünktlichkeit des Dozenten (Frage 1.2), die Übersichtlichkeit der Lehrmaterialien (Frage 1.3) und Bereitstellung der Lehrmaterialien (Frage 1.4). Drei dieser Fragen wurden von den Teilnehmern in den virtuellen Praktika signifikant häufiger mit „ja“ beantwortet als von den Teilnehmern in den in Präsenz durchgeführten Praktika (Frage 1.1: 100 % vs. 71,9 %; *p* < 0,001; Frage 1.2: 96,5 % vs. 45,9 %; *p* < 0,001; Frage 1.4: 90,7 % vs. 76,8 %; *p* < 0,01). Beim Vergleich der Antworten auf die Frage zur Übersichtlichkeit der Lehrmaterialien (Frage 1.3) zeigte sich hingegen kein signifikanter Unterschied (virtuell: 96,5 % „ja“ vs. Präsenz: 89 % „ja“; *p* = 0,14; Abb. 1a).

#### Themenbereich „Didaktik der Lehrveranstaltung“

Beim Vergleich der Evaluationsergebnisse für die Fragen zur Didaktik zeigte sich für alle vier abgefragten Aspekte eine signifikant bessere Bewertung für die virtuellen Praktika verglichen mit den Praktika im Präsenzformat. Die Fragen bezogen sich auf die Strukturierung der Lehrveranstaltung (2.1; virtuell: 97,7 % „ja“ vs. Präsenz: 82,2 % „ja“; *p* < 0,01), Darstellung von Lernzielen (2.2; virtuell: 95,3 % „ja“ vs. Präsenz: 78,7 % „ja“; *p* < 0,01), Wissenszuwachs im Verlauf der Lehrveranstaltung (2.3; virtuell: 97,7 % „ja“ vs. Präsenz: 88,6 % „ja“; *p* < 0,05) und das Hinweisen auf prüfungsrelevante Inhalte (2.4; 98,8 % „ja“ vs. 89,7 % „ja“; *p* < 0,05; Abb. 1b).

#### Themenbereich „Weitere Angaben zur Lehrveranstaltung“

In dieser Kategorie wurden weitere Kriterien evaluiert und zeigten in den folgenden Bereichen signifikant bessere Bewertungen für die virtuellen Praktika verglichen mit den Praktika unter Präsenzbedingungen: Wissenszuwachs durch den Besuch der Veranstaltung (3.1; virtuell: 92,0 % > 4 Punkte vs. Präsenz: 63,2 % > 4 Punkte; *p* < 0,001), Anschaulichkeit (3.2; virtuell: 93,1 % > 4 P. vs. Präsenz: 65,4 % > 4 P.; *p* < 0,001), Verständlichkeit (3.3; virtuell: 86,2 % > 4 P. vs. Präsenz: 65,2 % > 4 P.; *p* < 0,001), Relevanz (3.4; virtuell: 90,8 % > 4 P. vs. Präsenz: 78,4 % > 4 P.; *p* < 0,05), Struktur (3.5; virtuell: 93,1 % > 4 P. vs. Präsenz: 72,9 % > 4 P.; *p* < 0,001), Feedback (3.6; virtuell: 95,3 % > 4 P. vs. Präsenz: 69,0 % > 4 P.; *p* < 0,001) und wertschätzende Lernatmosphäre (3.7; virtuell: 96,4 % > 4 P. vs. Präsenz: 72,0 % > 4 P., *p* < 0,001). Lediglich bei den Antworten auf die Frage nach einer konstruktiven Lernatmosphäre durch Mitstudierende (3.8) ergaben sich keine signifikanten Unterschiede zwischen virtuellem und Präsenzformat (virtuell: 86,8 % > 4 P. vs. Präsenz: 79,6 % > 4 P.; *p* = 0,22; Abb. 2).

### Zusätzliche Evaluationsergebnisse für die digitale Lehre

Die beiden virtuellen Praktika (Wintersemester 2020/21 vs. Sommersemester 2021) wurden mit acht zusätzlichen Fragen evaluiert. Die Ergebnisse sind in Abb. 3 zusammengefasst. Die Frage nach der Zufriedenheit mit dem digitalen Lehrangebot (Frage 4.9) wurde von 87,5 % (Wintersemester 2020/21) bzw. 85,4 % der Teilnehmer (Sommersemester 2021) mit > 4 Punkten beurteilt.

In den offenen Feedbacks (*n* = 44) zu den beiden virtuellen Praktika fanden sich insgesamt *n* = 12 Aussagen mit dem Inhalt „Wunsch nach mehr Präsenzzeit/-unterricht“ (*n* = 3 im Wintersemester 2020/21; *n* = 9 im Sommersemester 2021).

### Vergleich der Evaluationsergebnisse für die beiden digitalen Semester

Bei keiner der Fragen zeigten sich beim statistischen Vergleich der Evaluationsergebnisse signifikante Unterschiede zwischen den beiden virtuellen Praktika (Wintersemester 2020/21 vs. Sommersemester 2021; Abb. 1, 2 und 3).

## Diskussion

Die universitäre Lehre befindet sich bereits seit längerer Zeit in einer Phase des Wandels. Im Mittelpunkt dieser Transformation steht dabei die Etablierung neuer, oftmals zumindest teilweise auf digitalen Technologien basierender Lehrformate und die verstärkte wissenschaftliche Auseinandersetzung mit innovativen didaktischen Konzepten [4, 5, 10, 16]. Diese Entwicklung trägt nicht zuletzt auch der Tatsache Rechnung, dass sich die Lern- und Arbeitsweisen der sog. Generation Y signifikant von früheren Generationen unterscheidet und insbesondere auch moderne Kommunikationstechnologien eine zunehmend große Rolle spielen [1, 6]. Dennoch war es erst der Beginn der SARS-CoV-2-Pandemie, der durch die plötzliche Notwendigkeit von Distanzunterricht die Lehre auch an den deutschen Hochschulen grundlegend verändert hat [11].

Mit dem Ziel der Kontaktreduktion während der SARS-CoV-2-Pandemie wurde an unserer HNO-Universitätsklinik das virtuelle Lehrkonzept des Interaktiv-Integrativ-digitalen HNO-Praktikums ab dem Wintersemester 2020/21 angewandt. Der Unterricht wurde dabei mit einem Videokonferenzsystem synchron online realisiert und beinhaltete lediglich eine kurze Präsenzphase (4 h) mit Patientenkontakt auf der Station und in der Hochschulambulanz.

Ein entscheidendes Messinstrument für die Qualität eines Lehrkonzepts stellt die studentische Evaluation dar, die vorrangig mit dem Ziel durchgeführt wird, die Qualität der Lehrveranstaltung auf Basis dieses Feedbacks zu verbessern [13]. Im Rahmen der im Fachbereich Medizin der Goethe-Universität Frankfurt langjährig etablierten Evaluationen von Lehrveranstaltungen wurde auch das HNO-Praktikum in jedem Semester durch die teilnehmenden Studierenden evaluiert. Mit durchschnittlich fast 30 % der Studierenden lag die Rücklaufquote der freiwilligen Evaluation etwas über dem Wert einer kürzlich veröffentlichten Untersuchung zur Evaluation von videobasiertem Distanzunterricht [14].

Das entscheidende und zugleich unerwartete Ergebnis dieser Untersuchung ist, dass die studentischen Evaluationen in nahezu allen abgefragten Kategorien signifikant bessere Ergebnisse für die digitale Lehre als für die Präsenzlehre erbrachten. Im ersten Fragenblock „Organisation der Lehrveranstaltung“ wurde zunächst der regelmäßige und pünktliche Beginn der einzelnen Lehrveranstaltungen signifikant häufiger bestätigt. Eine mögliche Erklärung hierfür ist, dass der Dozent bei einer virtuellen Lehrveranstaltung mit einem Videokonferenzsystem als Moderator die Session starten bzw. den Studierenden den Eintritt ermöglichen muss und für das Zustandekommen der Veranstaltung sowohl Lehrende als auch Studierende mehr zur Pünktlichkeit „gezwungen“ sind, als dies bei einer Präsenzveranstaltung der Fall ist. Auch scheint sich hier widerzuspiegeln, dass der in den verschiedenen Phasen der Pandemie reduzierte klinische Routinebetrieb mehr Raum für die ungehinderte Ausübung der Lehrtätigkeit schaffte, so wie auch eine gesteigerte Publikationsleistung deutscher HNO-Universitätskliniken in diesem Zeitraum beobachtet werden konnte [8].

Diese Einschätzung wird zusätzlich durch die Beobachtung gestützt, dass die in einzelnen Kategorien (z. B. Pünktlichkeit des Dozenten, Vermittlung schwieriger Inhalte, wertschätzende Lernatmosphäre) deutlich abfallenden Evaluationsergebnisse im Sommersemester 2019 in einem zeitlichen Zusammenhang mit der in dieser Phase kritischen ärztlichen Personalsituation in der Klinik stehen.

Da im Rahmen der Digitalisierung auch eine Reihe neuer Lehrmaterialien im Sinne einer „Modernisierung“ neu erstellt und online zur Verfügung gestellt bzw. präsentiert wurden, erklärt sich möglicherweise auch die verbesserte Bewertung durch die Studierenden in dieser Kategorie.

Alle vier Fragen zur Didaktik der Lehrveranstaltung wurden in den virtuellen Semestern signifikant besser evaluiert als in den Präsenzsemestern davor. Dies betraf die Struktur der Veranstaltung, das Formulieren von Lernzielen, den empfundenen Wissenszuwachs und das Hinweisen auf prüfungsrelevante Lehrinhalte. Dieses Ergebnis ist überraschend und ist nicht einfach zu interpretieren. Zwar wurden im Rahmen des virtuellen Praktikums zahlreiche neue Unterrichtselemente (Lehrvideos, Online-Audiometrieübungen usw.) eingeführt, allerdings erschließt sich zunächst nicht unmittelbar, warum die didaktischen Qualitäten des virtuellen Praktikums, das durch diese vermeintlichen „Online-Ersatzangebote“ geprägt war, von den Studierenden signifikant besser beurteilt wurden als im Präsenzpraktikum. Einen möglichen Hinweis könnten die von den Studierenden häufig geäußerten, sehr positiven Rückmeldungen zu der Unterrichtseinheit „HNO-ärztliche Operationen“ geben, denn es erscheint plausibel, dass diese synchron online kommentierten Videos von den Studierenden als lehrreicher empfunden werden als der frühere persönliche Aufenthalt im Operationssaal während des Präsenzunterrichts. Das Verfolgen z. B. mikrochirurgischer Eingriffe mag unter Präsenzbedingungen mitunter schwieriger sein und die Möglichkeit zum didaktisch anspruchsvollen Teaching durch den Operateur insbesondere in kritischen Operationsphasen nicht immer zu gewährleisten sein.

Lediglich bei zwei Fragen konnte kein signifikanter Unterschied zwischen virtuellen Praktika und Präsenzpraktika ermittelt werden. Diese Fragen betrafen die Übersichtlichkeit der Lehrmaterialien und den Beitrag von Mitstudierenden an einer konstruktiven Lernatmosphäre. Allerdings waren die für die virtuellen Praktika ermittelten Werte auch hier tendenziell besser als in den Präsenzpraktika.

Die Tatsache, dass die virtuellen Praktika von den Studierenden in nahezu allen Aspekten besser beurteilt wurden, könnte teilweise auch darin begründet sein, dass die Studierenden dankbar für ein Lehrangebot inmitten der pandemischen Situation waren und sich deshalb mitunter weniger kritisch zeigten. Eine Untersuchung von Offergeld et al. [12] bestätigt darüber hinaus, dass die aktuelle Generation der Studierenden gegenüber der Digitalisierung in der Medizin generell sehr aufgeschlossen ist. Dies spiegelt sich auch wider in der durchweg sehr positiven Beurteilung der spezifisch auf Aspekte des virtuellen Praktikums ausgerichteten Fragen (z. B. Frage nach Verhältnis Zeitaufwand zu Lernerfolg beim digitalen Lehrangebot).

Beim Vergleich der Ergebnisse der beiden virtuellen Praktika (Wintersemester 2020/21 und Sommersemester 2021) zeigten sich keine signifikanten Unterschiede. Daraus kann geschlossen werden, dass die positive Beurteilung durch die Studierenden kein kurzfristiges Ereignis darstellte, sondern dass das virtuelle Lehrformat zumindest in der Anfangsphase der Pandemie dauerhaft und wiederholt besser bewertet wurde. Allerdings darf nicht außer Acht gelassen werden, dass der Unterricht am Patienten weiterhin einen Grundpfeiler in der Ausbildung von Studierenden der Medizin darstellt [3] und nicht oder nur unzureichend in einem virtuellen Lehrformat abgebildet werden kann. Darauf deuteten auch zahlreiche individuelle Rückmeldungen der Studierenden vor allem im Verlauf des zweiten virtuellen Semesters hin, die zum Ausdruck brachten, dass der Mangel an Präsenzzeit/-unterricht in der Klinik zunehmend kritisch betrachtet wurde. Allerdings bleibt letztlich die Frage offen, ob sich diese Kommentare der Studierenden nur auf den Unterricht am Patienten oder auch auf andere Elemente/Aspekte des Präsenzunterrichts (z. B. praktische Übungen, persönlicher Kontakt zu Lehrpersonal und Kommilitonen) bezogen.

### Limitationen der Studie

Zunächst muss betont werden, dass es sich bei den für diese Untersuchung herangezogenen Evaluationsergebnissen um ein subjektives Feedback der Studierenden handelt und nicht um eine objektive Messung der Qualität der Lehrveranstaltung. Damit belegen die Daten zwar eine hohe Akzeptanz des virtuellen Unterrichts durch die Studierenden, allerdings lassen die Daten keine Rückschlüsse darauf zu, welchen Effekt die jeweilige Unterrichtsform auf den Lernerfolg (z. B. für die Vorbereitung auf das Staatsexamen im Fach HNO-Heilkunde oder die spätere ärztliche Tätigkeit) hat.

Bei der Betrachtung der Daten muss außerdem berücksichtigt werden, dass die vierstündige Präsenzphase (Station/Hochschulambulanz) als ergänzender Bestandteil des virtuellen Praktikums in die Evaluation miteinfloss und nicht gesondert bewertet wurde.

Einen weiteren möglichen Einflussfaktor für die Evaluationsergebnisse stellen wechselnde Dozenten während des Beobachtungszeitraums von 6 Semestern dar. Da jedoch in allen untersuchten Semestern bzw. Praktika innerhalb einer Praktikumswoche jeweils verschiedene Dozenten zum Einsatz kamen und das Evaluationsergebnis damit nie die Beurteilung eines individuellen Dozenten darstellt, kann auch hier davon ausgegangen werden, dass dieser Effekt zu vernachlässigen ist.

Die Teilnahme an der Evaluation erfolgte auf freiwilliger Basis. Aus diesem Grund kann nicht ausgeschlossen werden, dass Studierende mit hoher Motivation, Feedback (positiv oder negativ) zu geben, in der Auswertung überrepräsentiert sind. Dies würde bedeuten, dass die Ergebnisse nicht repräsentativ die Einschätzung aller Studierenden des jeweiligen Semesters widerspiegeln. Allerdings kommt dieser mögliche Effekt in allen Semestern in gleicher Weise zum Tragen, sodass hier nicht von einer „Verzerrung“ der statistischen Ergebnisse bei dem Vergleich der beiden Lehrformate (virtuell vs. Präsenz) auszugehen ist.

### Schlussfolgerungen

Aus den Ergebnissen dieser Arbeit lässt sich schließen, dass die Studierenden die Digitalisierung des Unterrichts insgesamt sehr positiv beurteilen. Diese Einschätzung zeigte sich über ein ganzes Jahr Pandemie konstant, wobei allerdings berücksichtigt werden muss, dass es sich mit der ersten Phase der Pandemie nur um einen begrenzten Beobachtungszeitraum handelte. Dass der virtuelle Unterricht gegenüber dem konventionellen Präsenzunterricht offensichtlich sogar als überlegen angesehen wird, scheint multifaktoriell bedingt zu sein und muss insbesondere angesichts des parallel dazu geäußerten Wunsches nach mehr Präsenzzeit in der Klinik differenziert betrachtet werden. Es erscheint plausibel, dass einzelne Unterrichtselemente (z. B. Demonstration von operativen Eingriffen) in einem digitalen Format unter Umständen besser vermittelt werden können als in Form eines klassischen Präsenzpraktikums. Daraus lässt sich der Schluss ziehen, dass für die Zeit nach der SARS-CoV-2-Pandemie mit Blick auf die Wünsche und Bedürfnisse der Studierenden ein hybrides Lehrformat im Sinne einer Präsenzveranstaltung mit gezieltem Einsatz ausgewählter virtueller Elemente eine geeignete Unterrichtsform darstellt und die Digitalisierung der Lehre weiter vorangetrieben und gefördert werden sollte.

Perspektivisch wird letztlich die systematische Aufarbeitung der Erfahrungen mit dem Einsatz digitaler Lehrkonzepte während der SARS-CoV2-Pandemie eine entscheidende Voraussetzung für die Entwicklung neuer, moderner Formate für die postpandemische Lehre darstellen. Die daraus resultierenden Erkenntnisse werden zeigen, in welchem Umfang virtueller Unterricht, insbesondere auch die Implementierung von innovativen digitalen Formaten wie z. B. Game-based Learning, Augmented Reality usw. [4] in Zukunft Teil des „new normal“ in der Hochschullehre sein sollte.

## Fazit für die Praxis

- Das virtuelle Format des HNO-Blockpraktikums (mit kurzer Präsenzphase) wurde von den Studierenden signifikant besser beurteilt als das konventionelle Präsenzformat.
- Die hohe Akzeptanz des virtuellen Unterrichts blieb in der Frühphase der Pandemie über die Dauer von zwei Semestern stabil, jedoch wurde gleichzeitig der Mangel an Präsenzzeit in der Klinik kritisch kommentiert.
- Die zukünftige (postpandemische) Lehre im Fach HNO-Heilkunde unter Präsenzbedingungen sollte durch virtuelle Unterrichtselemente ergänzt werden.
